# Simulation of heterogeneous tumour genomes with HeteroGenesis and *in silico* whole exome sequencing

**DOI:** 10.1093/bioinformatics/bty1063

**Published:** 2019-01-04

**Authors:** Georgette Tanner, David R Westhead, Alastair Droop, Lucy F Stead

**Affiliations:** bty1063-aff1 Leeds Institute of Medical Research at St James’s, St James’s University Hospital, Leeds, UK; bty1063-aff2 School of Molecular and Cellular Biology, University of Leeds, Leeds, UK; bty1063-aff3 Leeds Institute of Data Analytics, University of Leeds, Leeds, UK

## Abstract

**Summary:**

Tumour evolution results in progressive cancer phenotypes such as metastatic spread and treatment resistance. To better treat cancers, we must characterize tumour evolution and the genetic events that confer progressive phenotypes. This is facilitated by high coverage genome or exome sequencing. However, the best approach by which, or indeed whether, these data can be used to accurately model and interpret underlying evolutionary dynamics is yet to be confirmed. Establishing this requires sequencing data from appropriately heterogeneous tumours in which the exact trajectory and combination of events occurring throughout its evolution are known. We therefore developed HeteroGenesis: a tool to generate realistically evolved tumour genomes, which can be sequenced using weighted-Wessim (w-Wessim), an *in silico* exome sequencing tool that we have adapted from previous methods. HeteroGenesis simulates more complex and realistic heterogeneous tumour genomes than existing methods, can model different evolutionary dynamics, and enables the creation of multi-region and longitudinal data.

**Availability and implementation:**

HeteroGenesis and w-Wessim are freely available under the GNU General Public Licence from https://github.com/GeorgetteTanner, implemented in Python and supported on linux and MS Windows.

**Supplementary information:**

Supplementary data are available at *Bioinformatics* online.

## 1 Introduction

The evolution of a tumour affects its clinical course: malignant phenotypes undergo selection resulting in increased growth, invasion, metastases or therapy resistance. Elucidating the mode and course of tumour evolution therefore impacts upon our understanding of disease progression and how to more effectively treat patients (Amirouchene-Angelozzi *et al.*, 2017). Characterizing suitable models of tumour evolution and testing our ability to identify them from observable sequencing data requires datasets from tumours where the subclonal architecture and mutational events that define them are completely known; i.e. it requires sequencing data from realistically simulated, suitably heterogeneous tumours (Sun *et al.*, 2017; Williams *et al.*, 2016, 2018). Numerous tumour genome simulation tools exist (see Supplementary Material), but they lack the ability to recreate the complexity inherent in real data as they do not model certain phenomena known to occur through actual tumour evolution, such as: (i) multi-level subclone phylogenies, (ii) individual chromosome and whole-genome aneuploidy, (iii) overlapping copy number variants (CNVs)— either nested within the same chromosome copy, or partially or fully overlapping the same region on different copies, (iv) variants occurring in a flexible order and (v) distinct germline and somatic variants (see Supplementary Table S1).

To address these shortcomings, we developed HeteroGenesis: a simulator of genomes from realistically heterogeneous tumours that can result from varied and user-determined evolutionary trajectories. We also improved an *in silico* whole exome sequencing (WES) tool, Wessim (Kim *et al.*, 2013; McElroy *et al.*, 2012), and provide this as weighted-Wessim (w-Wessim). Together, these tools create sequencing data and ‘ground truth’ variant profiles for simulated bulk tumour samples, which can include ‘normal’ contamination and be manipulated to model multi-region and longitudinal sampling.

## 2 Implementation and workflow

### 2.1 HeteroGenesis

HeteroGenesis consists of three consecutive python programmes (Supplementary Box S1 and Fig. S1); ***heterogenesis_vargen*** generates lists of variants (single nucleotide variants [SNVs], insertions/deletions [InDels], CNVs [with or without inversion] and aneuploid events) to be incorporated into the genomes for each clone in a tumour, along with a matched germline. It takes as input: (i) a reference FASTA genome sequence, (ii) an optional file containing known germline SNV and InDel locations and minor allele frequencies formatted from dbSNP and (iii) a JSON file containing a set of parameters. It outputs a JSON file with lists of variants for each clone in the simulated tumour and a matched germline, as well as files containing the order that mutations occurred. ***heterogenesis_varincorp*** is run separately for each clone, and the germline sample. It takes the lists of variants generated by *heterogenesis_vargen* and incorporates them into the reference genome sequence, as well as calculating copy numbers and variant frequencies along the genome. ***freqcalc*** is then run to combine variant profiles for individual clones from *heterogenesis_varincorp* outputs to describe one or more bulk samples.

Further details are included in the Supplementary Material and Supplementary Figures S2–S4. HeteroGenesis is freely available at https://github.com/GeorgetteTanner/HeteroGenesis.

### 2.2 w-Wessim

We adapted the only existing *in silico* WES tool, Wessim (Kim *et al.*, 2013; McElroy *et al.*, 2012), to create w-Wessim, and combine it with an altered protocol. This includes, among others (see Supplementary Material), two significant improvements;

#### 2.2.1 Weighted probe selection

Wessim aims to mimic exome capture, during sequencing library preparation, through the use of BLAT (Kent, 2002) alignments of capture probe (primer) sequences to a genome in order to define regions for sequencing. However, the programme selects probes at random for *in silico* hybridization each time it creates a read, negating the modelling of copy number variation. We modified the code to weight probe selection by the number of times each probe aligns to a genome, thereby increasing the coverage in replicated regions.

#### 2.2.2 Probe sequences taken from real WES reads

The use of exome capture kit probes for *in silico* sequencing results in unrealistic read coverage distributions (Supplementary Fig. S5). Instead we provide a set of probes taken from reads in real WES data and show that this approach results in a more realistic distribution of generated reads.w-Wessim is freely available from https://github.com/GeorgetteTanner/w-wessim.

## 3 Discussion

### 3.1 HeteroGenesis

We developed HeteroGenesis to simulate genomes for multiple clones in a heterogeneous tumour, along with a matched germline. When compared with previous methods, HeteroGenesis has several significant improvements to allow it to recreate much of the complexity observed in real tumours (see Supplementary Material). In particular, the user has full control over the phylogenetic relationships between clones and, therefore, varied and complex evolutionary trajectories can be modelled (Supplementary Box S1).

Following tumour genome simulation, *freqcalc* can be used to combine variant information generated for each subclone to give overall bulk outputs that reflect user defined proportions of each clone in a sample. This is useful, for example, when testing how sampling affects the ability of subclonal deconvolution pipelines to elucidate different evolutionary trajectories from known ground truths. Moreover, this approach can: (i) include simulated germline sequencing data to mimic the contamination of normal cells in a tumour sample, (ii) create multiple bulk datasets from the same tumour with different proportions of clones to mimic multi-region sampling, an approach that enables more reliable delineation of subclone architectures (Sun *et al.*, 2017) and (iii) create multiple bulk datasets with different combinations of clones to represent longitudinal samples. An example of this is given in Supplementary Box 1.

### 3.2 w-Wessim

We sought a tool to enable *in silico* WES of our simulated genomes and were only able to identify Wessim for this purpose (Kim *et al.*, 2013; McElroy *et al.*, 2012). However, Wessim is not able to model copy number variation (Supplementary Fig. S5B), so we adapted it to create w-Wessim, which, through weighting probe selection by the number of times each is found to align to the genome, results in accurate modelling of copy number variation (Supplementary Fig. S5C).

We also aimed to improve the distributions of reads created by w-Wessim/Wessim to more realistically model WES data. Exon capture kits do not result in a perfect enrichment for only target regions, with only around 65% of reads aligning on-target (Supplementary Material). However, w-Wessim/Wessim result in very high proportions of bases aligning on or near target regions when used with probe sequences from the Agilent SureSelect Human All Exon V4+UTRs kit and default BLAT parameters; 90.6 and 90.0% on target and 99.6 and 98.1% on or near target for w-Wessim and Wessim, respectively (Supplementary Fig. S6A). Furthermore, the mode coverage in three real WES datasets that used the V5+UTRs kit, each with 70m reads, was 8x-29x, whereas the mode coverage for the same number of reads from w-Wessim and Wessim, using the V4+UTRs kit probes (the V5 probe sequences were not publically available), was 66x and 80x (Supplementary Fig. S6B).

We overcame this using read sequences from real WES data (with 75.8% of bases on or near target) as the probes in the BLAT alignment. By filtering these reads for those with a high alignment mapping score and adjusting the stringency of the BLAT alignment, we are able to generate reads with w-Wessim that match the distribution of reads seen in the real data, with 79.7% of bases aligning on or near target and a mode coverage of 28x (Supplementary Figs S5 and S6). A demonstration of the combined use of HeteroGenesis and w-Wessim is provided in Supplementary Figures S7 and S8.

## Supplementary Material

bty1063_Supplementary_Data
