# Supplementary material for: Simulation of heterogeneous tumour genomes with HeteroGenesis and in silico whole exome sequencing
Source: Bioinformatics. 2019 Jan 4;35(16):2850–2. doi: 10.1093/bioinformatics/bty1063 (PMC6691334; doi:10.1093/bioinformatics/bty1063)
Supplement: bty1063_Supplementary_Data [file bty1063_supplementary_data.pdf]

Simulation of Heterogeneous Tumour Genomes with HeteroGenesis and *In Silico* Whole Exome Sequencing

Georgette Tanner, David R Westhead, Alastair Droop and Lucy F Stead

Supplemental Information

1 Box 1 2

2 HeteroGenesis 3

2.1 Improvements over existing simulation methods 3

2.2 Workflow 7

2.2.1 *heterogenesis_vargen* 8

2.2.2 *heterogenesis_varincorp* 10

2.2.3 *freqcalc* 18

3 w-Wessim 18

3.1 Wessim 18

3.2 Improvements from Wessim 18

3.3 Requirements 24

3.4 Additional methods 25

4 Demonstration of HeteroGenesis with w-Wessim 25

1 Box 1

A stepwise example showing how HetergoGenesis and w-Wessim can be used to simulate spatiotemporal sampling from heterogeneous tumours.

Step 1

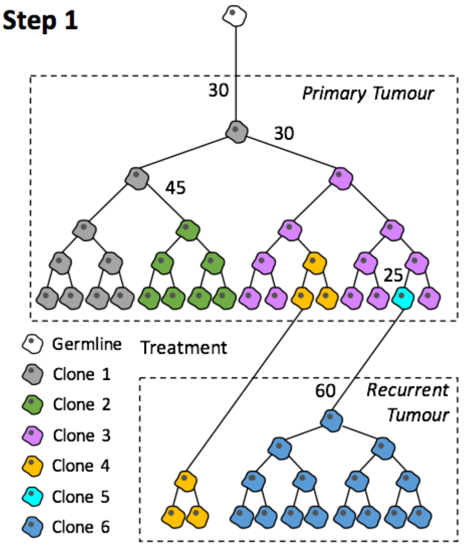

The user defines tumour evolution model. In this example a primary tumour (top) has arisen by neutral evolution. Following treatment, the majority of cells are eradicated but those that remain form a recurrent tumour (bottom) exhibiting big bang evolutionary dynamics. Circles denote cells and each cell division is shown. Cancer cells are coloured by genotype, i.e. distinct clones (numbered 1 to 6) are coloured differently. The numbers denote the amount of new mutations that have arisen during cell division to form a new clone.

Step 2

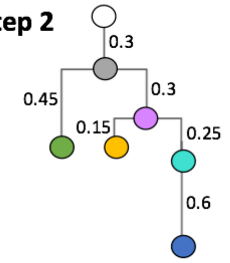

The user translates this into a phylogenetic tree and simulates this with HeteroGenesis. Here the example above has been translated into a phylogenetic tree showing the relationship between the germline genome and that of all 6 cancer clones, with evolutionary distances indicated. These parameters are input to ***heterogenesis_vargen*** which outputs lists of variants for the germline and each clone.

Step 3

HeteroGenesis creates genomes and variant profiles for the germline and each clone. The variant lists from step 2 are input to ***heterogenesis_varincorp*** to create the genome sequence (in FASTA format) and variant profile for the germline and each clone.

Step 4

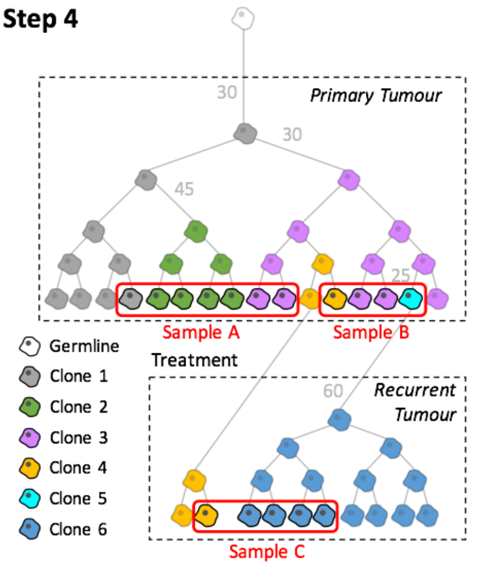

Sample A: 14% Clone 1, 57% Clone 2, 29% Clone 3
Sample B: 50% Clone 3, 25% Clone 4, 25% Clone 5
Sample C: 20% Clone 4, 80% Clone 6

The user decides what samples to acquire the bulk variant profiles and whole exome sequencing data for. Here three samples have been selected from the above example: two spatially distinct samples from the primary tumour and one from the recurrent tumour. The identity and proportion of clones in each sample are calculated. These can be scaled to include contamination by normal (germline) cells e.g. Sample C is 20% Clone 4 and 80% Clone 6; to model 25% contamination by normal cells in this sample, the adjusted proportions would be 25% Germline, 15% Clone 4 and 60% Clone 6.

Step 5

HeteroGenesis creates the variant profile for each sample. Clone and proportion information from step 4 is input to ***freqCalc*** which outputs the variant profiles (i.e. ground truths) for each sample.

Step 6

w-Wessim creates the sequencing data for each sample. The genome sequence and proportion-based number of reads for each clone (or the germline) is input to w-Wessim. This outputs FASTQ files which can be combined to create sample-level sequencing data.

2 HeteroGenesis

2.1 Improvements over existing simulation methods

Methods for investigating the subclonal architecture and progression of tumours from genome sequencing data require testing on realistically complex simulated datasets. However, existing simulation tools lack the ability to model certain scenarios frequently observed in real tumours (Supplementary Table 1), but which are included in HeteroGenesis. These include:

i. **Multi-level subclone phylogenies:** Subclones in tumours have complex phylogenetic architectures(Sottoriva *et al.*, 2013; McPherson *et al.*, 2016; Watkins and Schwarz, 2018) Current simulation tools create: no subclones(Hosny, 2017; Mu *et al.*, 2015a), single layer phylogenies(Qin, Liu, Conroy, Morrison, Hu, *et al.*, 2015; Xia *et al.*, 2017), or hierarchical structures but with no access to intermediate level genomes(Ivakhno *et al.*, 2017). More complex phylogenies can be created through iterative running of these tools, however this creates issues with keeping track of variant positions with respect to a stable reference. Xome-Blender(Semeraro *et al.*, 2018) SVEngine(Xia *et al.*, 2018) and the ICGC-TCGA DREAM BAMSurgeon wrap around script(Salcedo *et al.*, 2018) are exceptions to this, being able to create complex phylogenies, but instead succumb to other points mentioned below.

   With HeteroGenesis, the user has full control over the subclonal architecture of tumours by defining two parameters per clone: parent clone and the evolutionary distance from it. Varied and complex evolutionary trajectories can therefore be modelled.

ii. **Individual chromosome and whole-genome aneuploidy:** Both individual chromosome and whole-genome aneuploid events are common in cancer(Baysan *et al.*, 2017; Hu *et al.*, 2013) but are not included in many existing tumour genome simulators, particularly for individual chromosomes.

HeteroGenesis simulates a user defined number of aneuploid events, with a user defined probability that each will be for a single chromosome or the whole genome. New copies of chromosomes inherit the existing variants on the parent chromosome, and then acquire further variants unique to each copy.

iii. **Overlapping copy number variants (CNVs):** Given that tumours contain numerous CNVs, often reaching 10s of megabases in length(Krijgsman *et al.*, 2014; Tan *et al.*, 2014) it is likely that many will overlap, either i) nested within the same copy of a chromosome, ii) partially or fully on different copies within the same cell, or iii) partially or fully on copies in separate subclones. Many existing simulation tools do not allow for this.
In HeteroGenesis, overlapping CNVs are made possible by splitting the genome at every breakpoint into blocks that can be sequentially replicated or removed with each CNV.

iv. **Variants occurring in a flexible order:** Real genomes acquire different types of variants in a flexible and varied order. As a result, a single nucleotide variant (SNV) may appear in only one, or in multiple copies of a replicated region, depending on whether it occurred before or after a copy number variant (CNV) or aneuploid event. However, existing tools incorporate different types of variants in separate stages. For example, Pysim-sv(Xia *et al.*, 2017) generates all aneuploid events prior to SNVs, and all SNVs prior to CNVs. Therefore, aneuploid copies of a chromosome in a clone won't share any common variants, and SNVs will always be present in every copy of an overlapping CNV region on a chromosome.
In order to accommodate flexible orders of variant incorporation by HeteroGenesis, the number of occurrences of each SNV and InDel in a genome are calculated from CNVs that occur subsequently over each variant. This requires keeping track of CNV break points to determine whether a new CNV falls within or around an existing CNV, and therefore how many existing copies should be multiplied by the new CNV copy number.

v. **Distinct germline and somatic variants:** The majority of SNVs and insertions and deletions (InDels) in human germline genomes are at known polymorphic loci(1000 Genomes Project Consortium *et al.*, 2015), recorded in dbSNP(Sherry *et al.*, 2001). Some somatic SNV callers make use of this information in determining the confidence with which an apparently tumour specific variant is assigned as somatic(Fan *et al.*, 2016; Cibulskis *et al.*, 2013). Such callers will be biased against when applied to simulated germline genomes without a proportion of variants at polymorphic loci. Likewise, the approach used by Xome-Blender(Semeraro *et al.*, 2018), which simulates tumour genome sequencing reads by re-assigning true germline variants as somatic, would result in biased metrics for SNV calling. Other simulation tools do not include somatic SNVs at all(Qin, Liu, Conroy, Morrison, Hu, *et al.*, 2015; Xia *et al.*, 2018).

With HeteroGenesis, the user has the option to take a proportion of the germline SNVs and InDels from known variants in dbSNP and, unlike any previous method, weights them by their frequency in the population.

Furthermore**,** some existing somatic genome simulators(Semeraro *et al.*, 2018; Ivakhno *et al.*, 2017; Ewing *et al.*, 2015; Salcedo *et al.*, 2018) incorporate somatic variants directly into real sequencing data, as opposed to generating genome sequences. While this avoids the need for *in silco* sequencing, the effect that variants have on library preparation, particularly in WES probe hybridisation, is not taken into account. In addition, further problems are introduced due to the ground truth of existing variants in the real data not being known, as well as being limited in the copy number of simulated CNVs by the coverage depth of the inputted data.

One such method is BAMSurgeon. The ICGC-TCGA-DREAM team have used this tool to create datasets for crowd-sourced benchmarking of subclonal deconvolution methods in their ‘Somatic

Mutation Calling Challenge --Tumor Heterogeneity and Evolution’. For this, they developed a wrap around script for BAMSurgeon that adds features such as including aneuploid events(Salcedo et al., 2018). However, we found it difficult to find sufficient details to determine if it enables certain other features, such as overlapping CNVs. In addition, BAMSurgeon has a known issue in that SNVs/InDels in CNV regions tend to have inaccurate VAFs and therefore it is recommended to mask those regions from variant calling. This impairs its suitability for use in testing methods that investigate tumour evolution.

Supplementary Table S1. Features modelled by existing somatic simulation tools. ‘X?’ indicates that the feature is not mentioned in the accompanying documentation and we could find no evidence of it within the programme, so is highly likely not to be included.

Feature	**SCNVSim** (Qin, *et al.*, 2015)	**VarSim** (Mu *et al.*, 2015b)	**tHapMix** (Ivakhno *et al.*, 2017)	**Pysim-sv** (Xia *et al.*, 2017)	**Xome-Blender** (Semeraro *et al.*, 2018)	**SVEngine** (Xia *et al.*, 2018)	**BAMSurgeon wrapper** (Salcedo *et al.*, 2018)
Multi-level phylogenies	X	X	X	X	✓	✓	✓
Flexible variant order	X	X?	X?	X	X?	X?	✓
Overlapping CNVs	X	X?	X?	X	X?	X?	X?
Individual chrom & genome aneuploidy	✓	X?	✓	✓	X	✓	✓
Distinct germline and somatic SNVs/InDels	X	✓	✓	✓	X	X?	✓
Generates genome sequences	✓	✓	X	✓	X	✓	X

2.2 Workflow

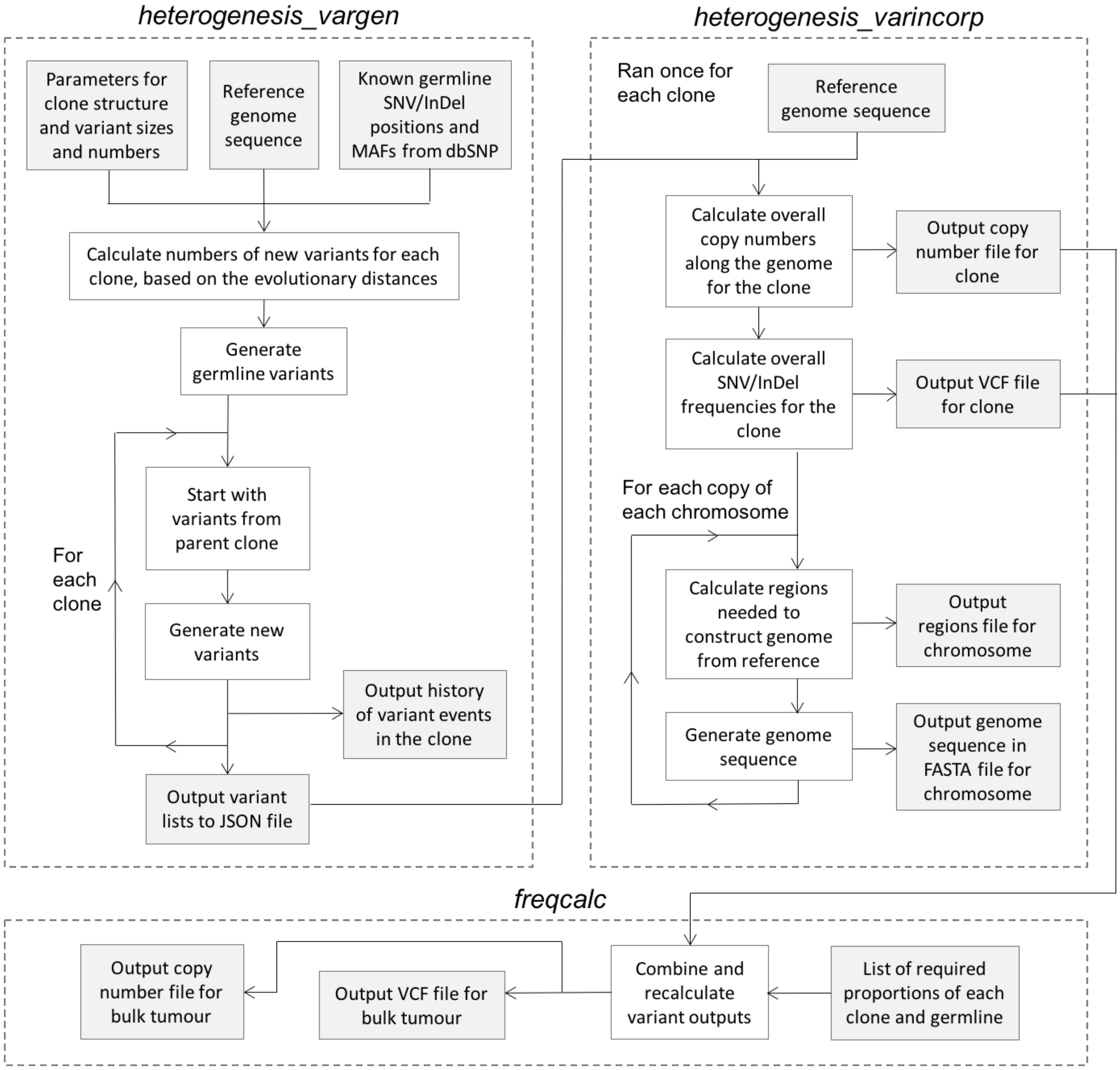

Supplementary Figure S1. The workflow of HeteroGenesis. *heterogenesis_vargen* first generates lists of variants for the germline and each somatic clone in the tumour. *heterogenesis_varincorp* then incorporates these variants into a reference genome and calculates variant frequencies and copy numbers along the genome for a given clone. *freqcalc* can then be used to calculate overall bulk tumour variant profiles.

An overview of the HeteroGenesis workflow is provided here, with more detailed information on the coding logic available at https://github.com/GeorgetteTanner/HeteroGenesis.

HeteroGenesis consists of three consecutive python programs:

2.2.1 *heterogenesis_vargen*

heterogenesis_vargen generates lists of variants (single nucleotide variants (SNVs), insertions/deletions (InDels), CNVs and aneuploid events) to be incorporated into the genomes for each clone in a tumour, along with a matched germline. It takes as input: i) a reference FASTA genome sequence, ii) an optional file containing known germline SNV and InDel locations and minor allele frequencies formatted from dbSNP, and iii) a JSON file containing a set of parameters.

It outputs a JSON file with lists of variants for each clone (herein also referring to the matched germline, which is considered the germline 'clone') in the simulated tumour, as well as files containing the order that mutations occurred. The user is able to define the: i) subclonal structure, ii) number of somatic aneuploid events, iii) rates of SNVs and InDels, iv) length distributions of InDels, and v) number and length distributions of CNVs. Separate parameter values are set for germline and total somatic variants. Users can also choose whether, and to what extent, to incorporate known germline variation into the simulated germline genome, weighted by minor allele frequency.

The clone structure of a tumour is defined by giving, for each clone (C_i), its direct parent clone and a value representing the evolutionary distance from it (D_i). These values are used to determine what proportions of the total somatic variants, (T), are assigned as new variants in each clone, thereby reflecting how far a clone has evolved from its parent. Therefore the number of new somatic variants in a clone, C_i, are defined by $\mathrm{T}\frac{\mathrm{D_i}}{\sum_1^n \mathrm{D_i}}$. This allows the user full control over the mode of evolution in each tumour. (See Box 1.)

CNV (>50 bases) and InDel (≤50 bases) lengths follow scaled log normal distributions, which have been observed in real data from both ours and other groups(Droop *et al.*, 2018; Krijgsman *et al.*, 2014), with user defined parameters for the mean and variance of the underlying normal

distribution, and a scaling factor. All default values for variant parameters are chosen to reflect estimates from real human germline(Mills *et al.*, 2006; 1000 Genomes Project Consortium *et al.*, 2015; Durbin *et al.*, 2010) and tumour genomes (specifically from glioblastoma)(Baysan *et al.*, 2017; Hu *et al.*, 2013; Kandoth *et al.*, 2013; Krijgsman *et al.*, 2014; Xi *et al.*, 2011).

The program first determines the total numbers of each type of somatic variant required in the final tumour and randomly splits these between somatic clones, based on the evolutionary distances between them from the provided parameters. Variants are then generated for each clone, starting with the germline clone. Each clone is initiated with all the variation inherited from its parent clone (with the root clone inheriting variation from the germline) and new variants are then added in a random order with respect to variant type. Only the following are disallowed for pragmatic reasons: i) SNVs and InDels cannot occur more than once at a base on the same copy of a chromosome in a clone, ii) CNVs or InDel deletions cannot partially overlap on the same copy of a chromosome in a clone (fully overlapping on the same chromosome, or partially/fully overlapping on different copies of a chromosome, can occur), and iii) no variant can occur within a deleted region, even if there are additional copies of the region on the chromosome (from a CNV) that haven't been deleted (though these may contain variants that precede the deletion). Chromosomes are selected at random for placing variants, taking length into account, except for aneuploid events where all chromosomes are selected with equal probability. Variants are initially placed on either of two sets of chromosomes, thereby simulating a diploid genome. However, after an aneuploid replication event has occurred, additional copies of that chromosome, containing the same set of existing variants, are then available for further variants to be incorporated. Similarly, when a deletion aneuploid event has occurred, the deleted chromosome is no longer available for variant placement and is not written to the outputs.

heterogenesis_vargen takes 2hrs and 4GB RAM on a single thread to run under default parameters, which includes a germline and 2 somatic clones.

2.2.2 *heterogenesis_varincorp*

heterogenesis_varincorp is run separately for each clone. It takes the lists of variants generated by *heterogenesis_vargen* and incorporates them into a reference genome sequence, as well as calculating copy numbers and variant frequencies along the genome. This is done by sequentially using the variants in the list to update three items:

ii. **cnblocks.** Lists of chromosomal regions (herein referred to as blocks) for each copy of a chromosome, used to calculate copy numbers. Each list is initiated with a single block equal to the length of the chromosome. It is updated each time a new CNV is incorporated by splitting blocks at CNV breakpoints and either replicating all blocks within the breakpoints (CNV replication) or removing them (CNV deletion). As direction is not relevant to copy number calculations, inversion information is ignored. After all variants have been incorporated, the number of blocks in all copies of a chromosome that correspond to each region are combined. This gives the overall copy number status along each chromosome, which is then written to a tab-delimited file. (Supplementary Fig. S2)

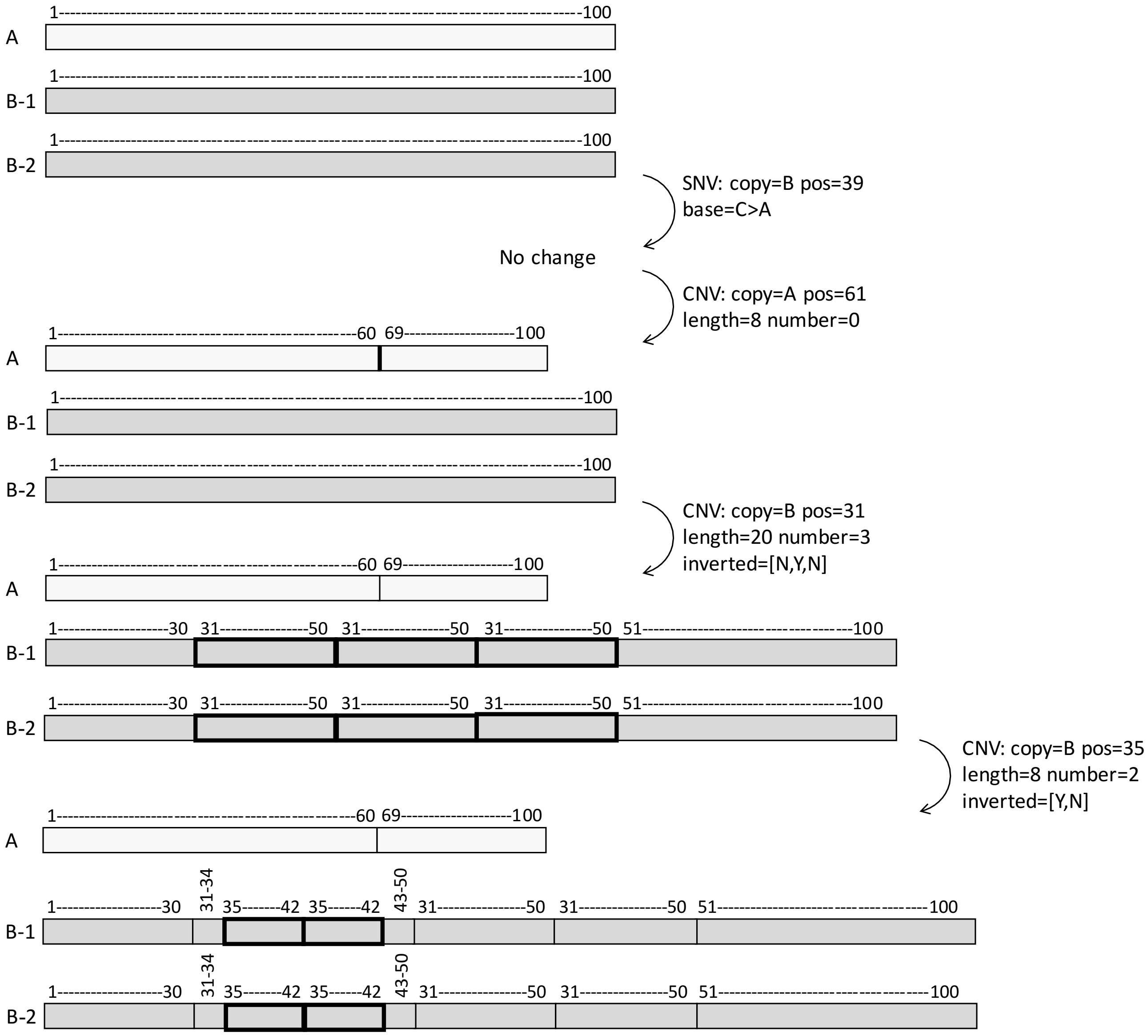

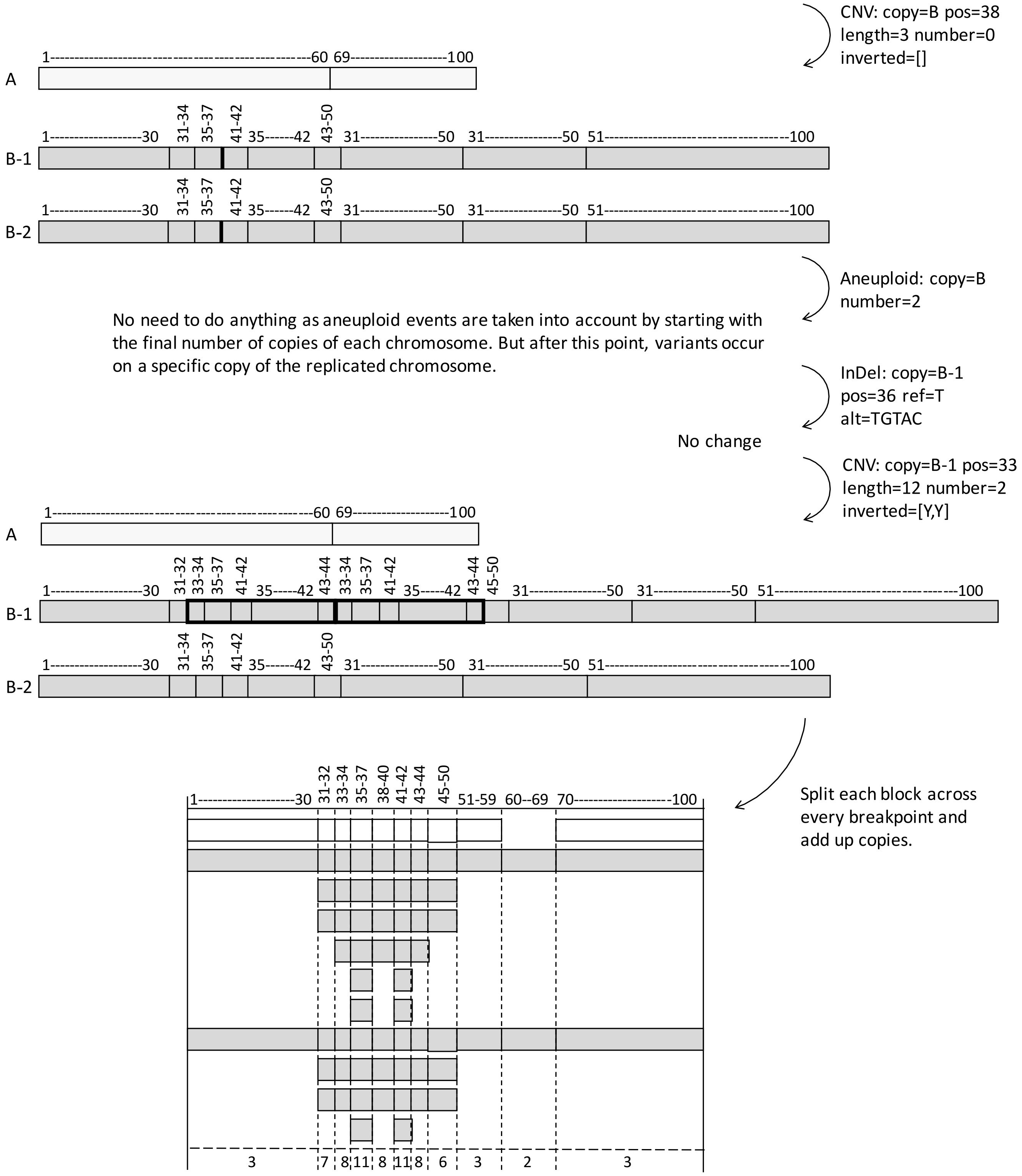

Supplementary Figure S2. An illustration of how cnblocks is updated and used to calculate copy number along a chromosome given a list of variants.

iii. **allblocks**. Analogous to cnblocks, but also includes blocks representing SNVs and InDels, and flags for starts and ends of inverted regions are recorded. The genome sequence for each copy of each chromosome is generated using the allblocks lists, which act as blueprints for constructing the genome sequences from the reference sequence. For each block in allblocks, the genome sequence is extended with either the corresponding reference sequence at the given positions or the alternate allele sequence. When an inversion start flag appears, the succeeding sequence is held separately until an end flag appears, at which point the held sequence is inverted, translated into the complimentary sequence, and added onto the main sequence, or to a previously held sequence if there is an overlapping inverted sequence. After all blocks for a chromosome have been passed, the sequence is written to a FASTA file. (Supplementary Fig S3)

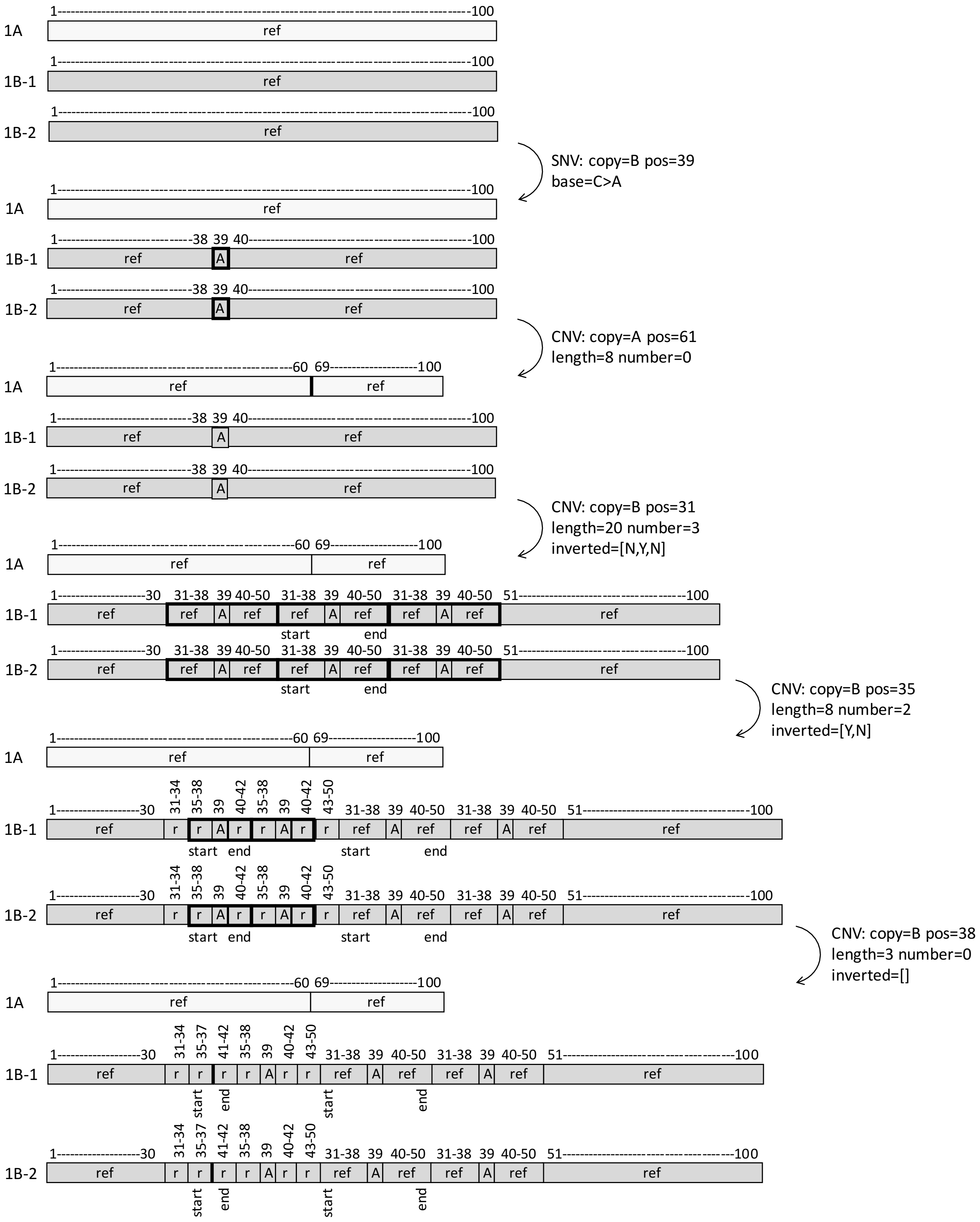
1A
1B-1
1B-2
ref
SNV: copy=B pos=39
base=C>A
CNV: copy=A pos=61
length=8 number=0
CNV: copy=B pos=31
length=20 number=3
inverted=[N,Y,N]
CNV: copy=B pos=35
length=8 number=2
inverted=[Y,N]
CNV: copy=B pos=38
length=3 number=0
inverted=[]
start
end

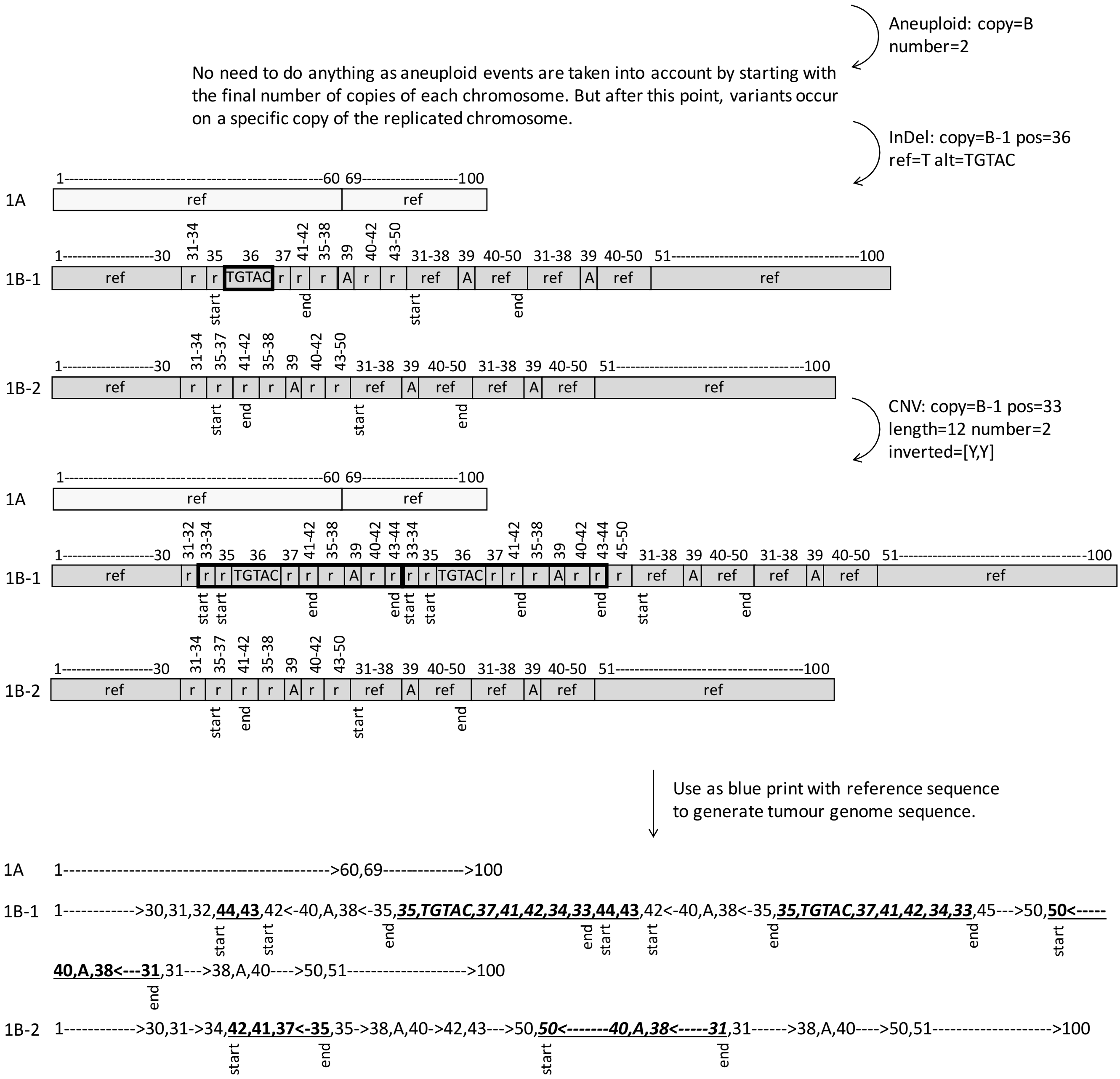

Supplementary Figure S3. An illustration of how allblocks is updated and used to generate a FASTA sequence given a list of variants. Bold boxes indicate the latest variant incorporation. 'start' and 'end' flags indicate where inversions occur. Underlined bold sequences indicate regions that have been inverted, where the complementary base sequence will be written in the FASTA file. Regions that have been inverted twice, from overlapping inversions, are written in the forward direction.

iv. **vcfcounts**: Lists of incorporated SNVs and InDels for each copy of a chromosome, with the number of occurrences recorded for each. Each SNV/InDel also has information recorded on the position of CNVs that overlap them. This enables calculation of how many occurrences of an SNV/InDel to replicate/remove based on whether a new CNV falls within or around a previous CNV. Once all variants have been incorporated, the vcfcounts list for all copies of a chromosome are combined, with shared SNV/InDels' numbers of occurrences added together. The overall copy number at each SNV/InDel position is taken from the combined cnblocks list and used with the total number of occurrences to calculate variant allele frequencies. These are then written to a variant call format (VCF) file. (Supplementary Fig. S4)

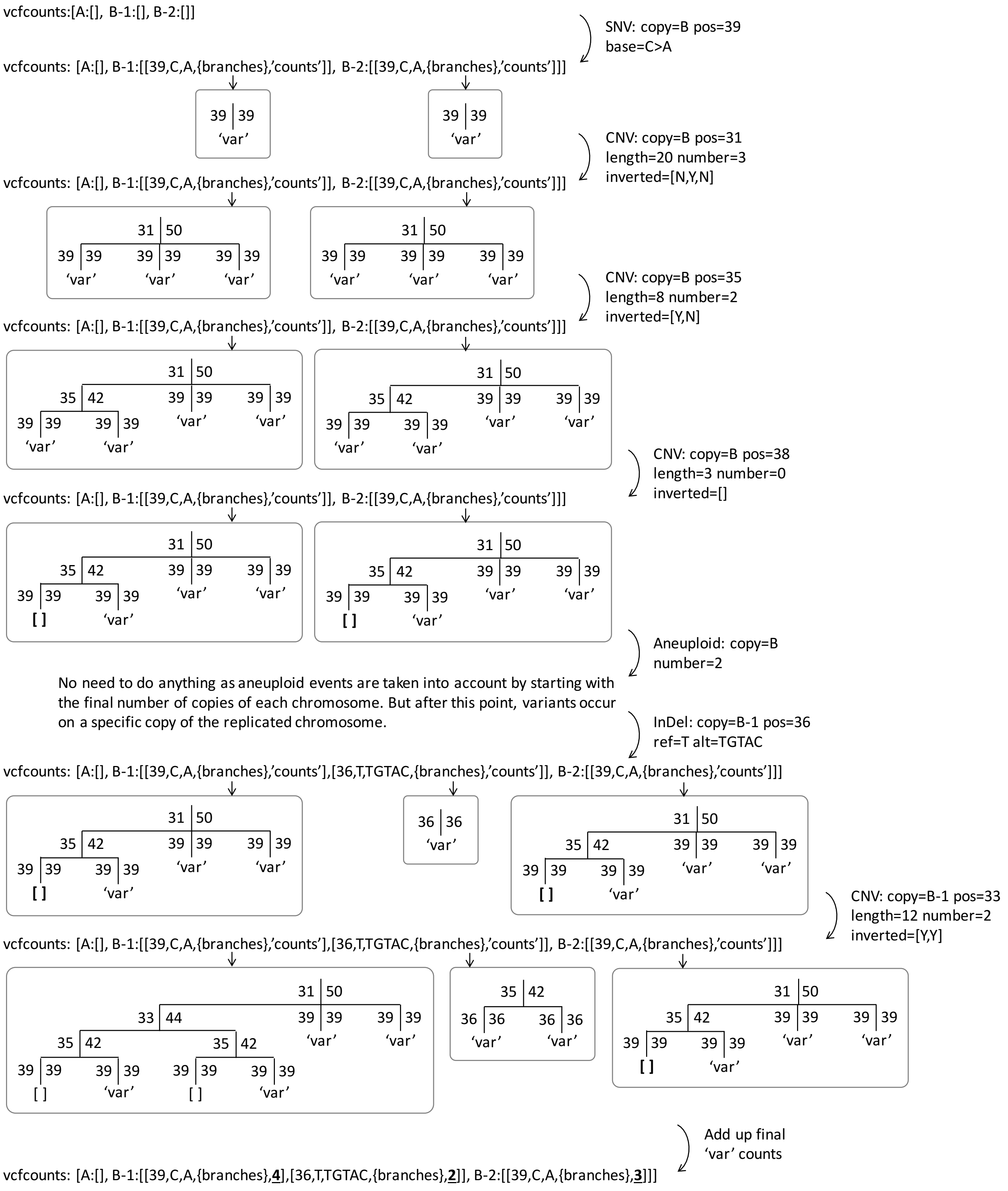

Supplementary Figure S4. An illustration of how vcfcounts is updated and used to calculate VAFs given a list of variants. The tree diagrams represent the information that is contained in the 'branches' slot for each variant listed in vcfcounts.

Downward lines represent a copy of the region, with values giving the start and end positions. Each succeeding level shows either the presence of one variant (‘var’), an absence of a variant from a deletion (‘[ ]’), or the CNVs contained within the above region.

heterogenesis_varincorp takes 1hr and 7GB RAM on a single thread to run ’clone1’ of the output from *heterogenesis_vargen* ran under default parameters.

2.2.3 *freqcalc*

freqcalc is provided as an accessory tool within HeteroGenesis and is used to calculate overall bulk tumour variant profiles. It takes the VCF and copy number outputs for each clone from *heterogenesis_varincorp*, along with a file specifying proportions of each somatic clone and the germline in a sample. *freqcalc* then calculates and outputs equivalent information for a bulk tumour that contains the given clone proportions.

3 w-Wessim

3.1 Wessim

Wessim(Kim *et al.*, 2013) is an *in silico* WES tool that combines fragment selection from target regions with the whole genome *in silico* sequencing tool, GemSIM(McElroy *et al.*, 2012). Target regions are determined through a Blat(Kent, 2002) alignment of exon capture hybridization probe sequences to a genome. Selected fragments are taken from these regions and filtered based on length and GC-content to reproduce realistic biases. These are then used to generate sequencing reads from, while incorporating realistic error rates, by a module from GemSIM.

3.2 Improvements from Wessim

We adapted Wessim to create weighted-Wessim (w-Wessim), and combine it with an altered protocol. Together these allow:

i. **Weighted probe selection.** Wessim aims to mimic exome capture, during sequencing library preparation, through the use of BLAT(Kent, 2002) alignments of capture probe (primer) sequences to a genome in order to define regions for sequencing. However, the program selects probes at random for *in silico* hybridisation each time it creates a read, negating the modelling of copy number variation. We modified the code to weight probe selection by the number of times each probe aligns to a genome, thereby increasing the coverage in replicated regions.

ii. **Probe sequences taken from real WES reads.** The use of exome capture kit probes for *in silico* sequencing results in unrealistic read coverage between targeted and off-target regions (Supplementary Fig. S5-S6). The Agilent SureSelect Human All Exon V5+UTRs kit is estimated by the manufactures to capture reads with only approximately 65% aligning to target regions and 77% aligning ± 100bp. Additionally, three WES datasets from the NCBI Sequence Read Archive, that had been created independently with this kit and sequenced by an Illumina HiSeq 2500, were found to have 56.5%-61.0% of bases aligning on, and 67.9%-77.3% bases aligning on or near target regions (Supplementary Fig. S6). Furthermore, when visualising alignments of these real reads, a background level of off target reads is seen between the larger (and generally on target) peaks (Supplementary Fig. S5A). However, using the probe sequences for the V4+UTRs exon capture kit (the most recent kit for which the sequences have been made available, and estimated to have 80% reads aligning on target and 86% aligning on target ± 100bp) with w-Wessim/Wessim, resulted in very high proportions of bases aligning near to, or on, target regions; 90.6% and 90.0% on target and 99.6% and 98.1% on or near target for w-Wessim and Wessim respectively (Supplementary Fig. S5-S6). In addition, the mode coverages for the three real WES datasets, when

subsampled down to 70m reads, were 8x, 28x and 29x, whereas the mode coverages for the same number of reads generated by w-Wessim and Wessim, was 66x and 80x, respectively (Supplementary Fig. S6B).

We instead provide a set of 101,846,922 probes taken from 99bp reads in real WES data from the Sequence Read Archive (SRR2103613 - frozen normal adult male lung, 61.0% and 75.8% of bases on, and on or near, target respectively) as the probes in the BLAT alignment. This dataset had the median percentage of on target reads of the three datasets we found that were created with the Agilent V5+UTRs kit and Illumina HiSeq 2500. Filtering the reads for those which aligned with a mapping score of 60 in paired-end mode by BWA-MEM(Li, 2013) to the Hg38 reference genome, was needed to prevent too many off-target bases. However, after converting these to single end reads with a top quality score for every base, only 96.24% of reads had a mapping quality of 60, which is similar to the proportion of probes in the V4+UTRs exon capture kit that have a mapping quality of 60 (96.54%).

We also increased the stringency of the BLAT from default parameters by increasing the minimum score and minimum percentage identity for alignments, both to 95. This further reduced the number of off-target reads, and is also likely to more accurately reflect real exon capture hybridisation in the lab, thereby resulting in more realistic modelling of the affect that variants have on exon capture. The final proportion of on, and on or near, target bases generated by w- Wessim, using the filtered real data reads and increased stringency BLAT alignment, was 61.1% and 79.7% respectively, and with a mode coverage of 28x for 70m reads (Supplementary Fig. S5D and S6).

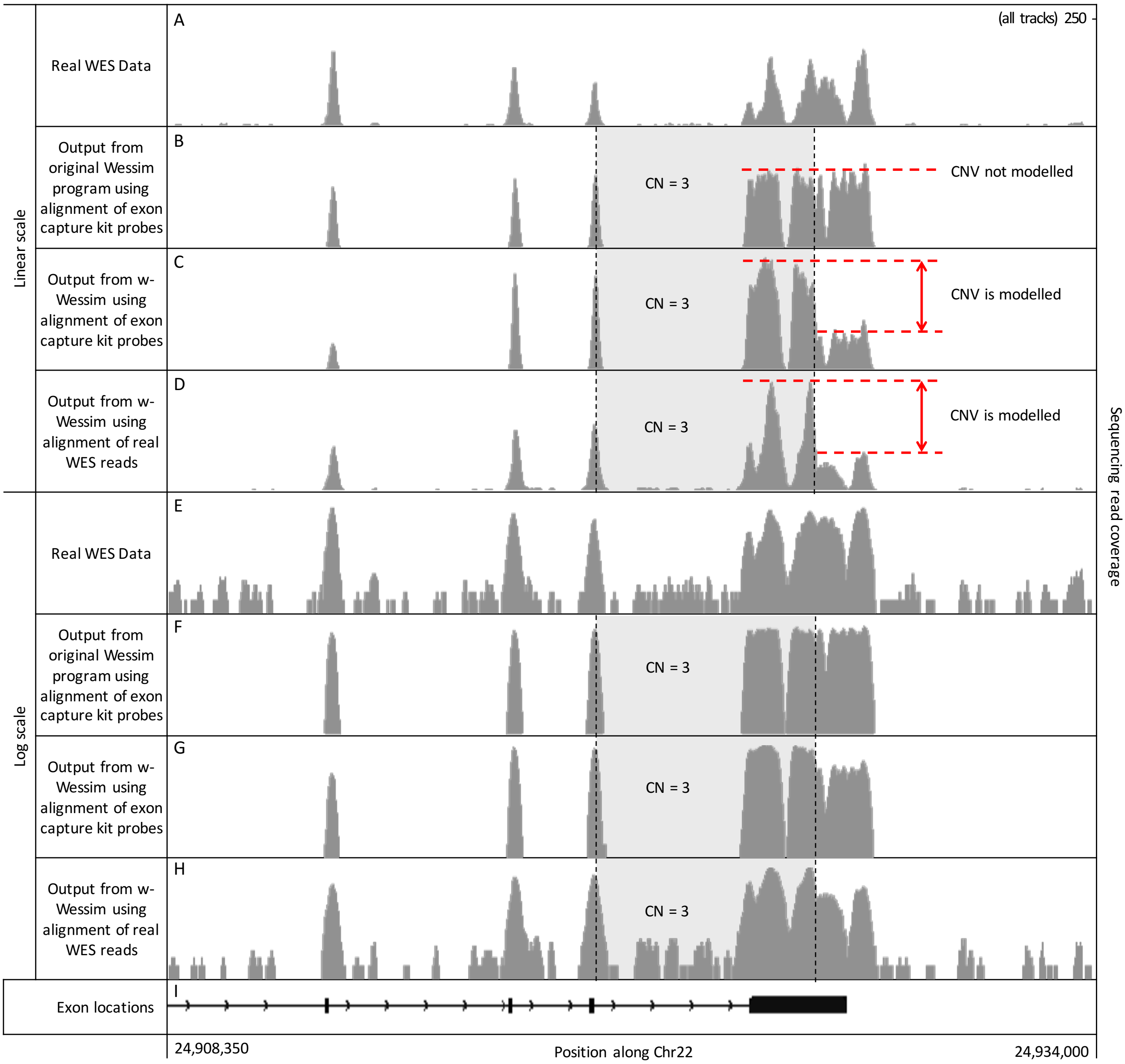

Supplementary Figure S5. Distributions of real and simulated WES reads along a region of chromosome 22, with linear scales of coverage depth (A to D, enabling copy numbers to become apparent) and log scales (E to H, enabling off target coverage to become apparent). Simulated data was generated from the hg38 human reference genome that had a CNV with a copy number of 3 inserted at position chr22:24,917,701-24,926,065. A+E) Real reads from the SRR2103613 data set. B+F) Reads generated by the original Wessim program using the recommended protocol with a BLAT alignment of Agilent SureSelect Human All Exon V4+UTRs

kit probe sequences. C+G) Reads generated by w-Wessim using the original Wessim recommended protocol with a BLAT alignment of Agilent SureSelect Human All Exon V4+UTRs kit probe sequences. D+H) Reads generated by w-Wessim using our modified protocol with a BLAT alignment of real reads from the SRR2103613 data set. I) Position of exon and intron locations, shown as boxes and lines respectively.

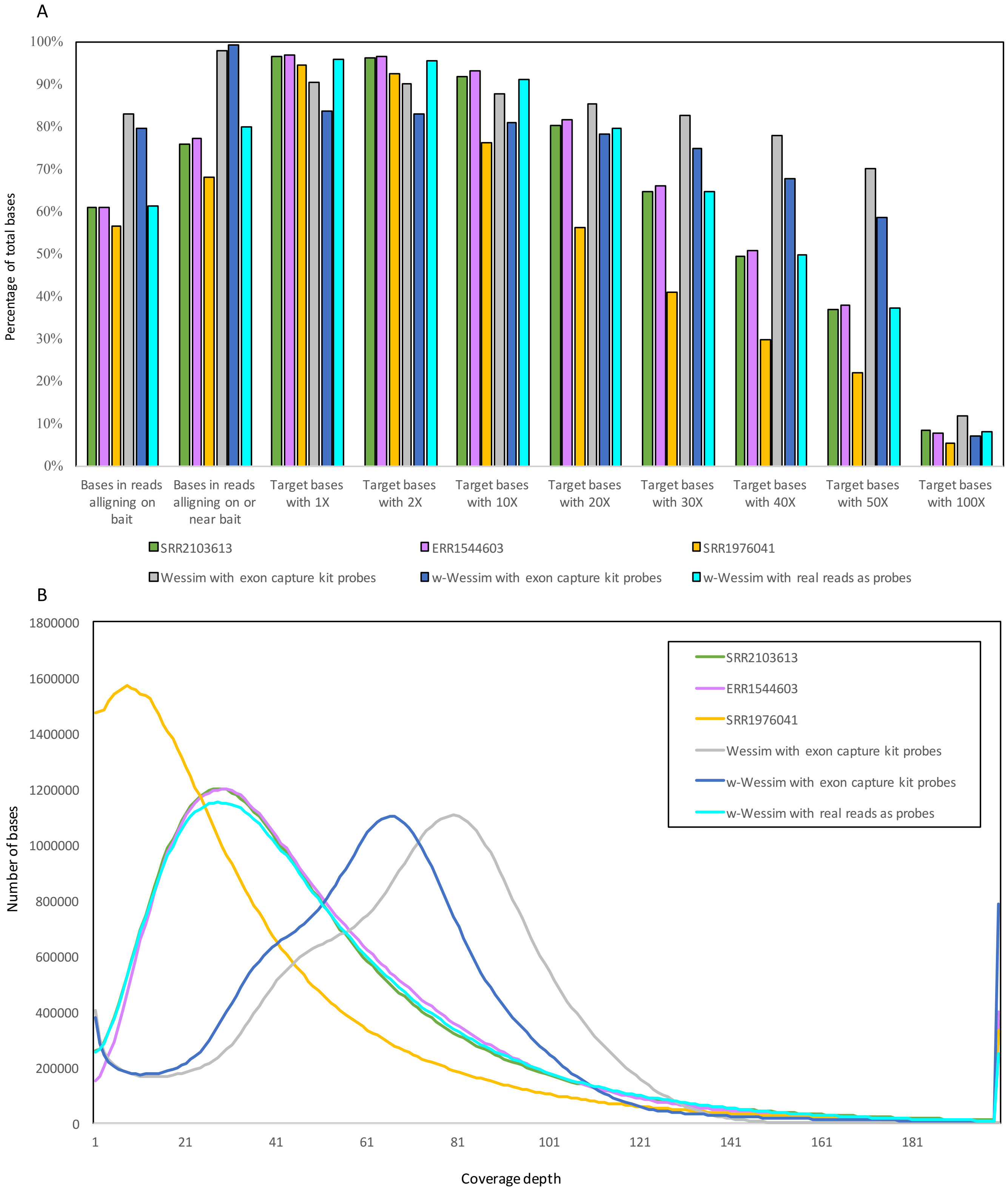

Supplementary Figure S6. Coverage metrics for three publically available real WES datasets (named by their SRA accession number) created with the Agilent SureSelect Human All Exon V5+UTRs kit, and three simulation methods that use

either the Agilent SureSelect Human All Exon V4 kit probe sequences or real reads from the SRR2103613 dataset as probes. All datasets contained 70m reads.

iii. **Read lengths to be taken from a distribution, instead of a fixed length.** Wessim uses error models trained by GemSIM(McElroy *et al.*,2012) on aligned real sequencing reads to guide error incorporation. Quality and adapter trimming of the training reads allows for a more accurate alignment, however this means that many errors (which tend to be at higher frequencies towards the end of reads) will have been removed and not incorporated into the error model. This can be taken into account by modelling the effect of trimming through simulating reads that follow the same length distributions as the trimmed training set. We therefore allow w-Wessim to use the read length distributions in the GemSIM error model; this was achieved using code taken from GemSIM.

iv. **Generation of sequencing fragments with a length distribution that, in some cases, falls below the specified read length.** We wished to allow modelling of formaldehyde-fixed, paraffin embedded (FFPE) samples, which generally contain shorter fragment lengths. However, lowering the length distribution for fragments in Wessim can results in lengths being selected that fall below the chosen read lengths. We therefore modified the code to handle such scenarios when they occur in w-Wessim.

3.3 Requirements

w-Wessim takes 9h/threads and 72GB RAM x threads to generate 1×10^7 pairs of reads when using probes taken from real WES reads. The large memory requirement results from the high number of real reads used as probes, which can be downsampled if needed. A BLAT alignment of the probes to the genome being sequenced is necessary prior to running w-Wessim. For the full set of $1x10^8$ probes used in our example, this would take ~700h/threads. However, this can be ran across multiple nodes in separate runs by splitting the read number, probes or genome sequence.

Alternatively, probe sequences for the exome capture kits can be used to significantly speed up runtime and reduce the memory requirement. However, this does not result in the realistic read distribution seen when using probes taken from real WES reads.

3.4 Additional methods

Exome sequencing metrics, used to compare our simulated sequences with real data, were assessed using Picard HsMetrics(Broad Institute) with the 'Covered.bed' target files downloaded from the Agilent website (https://earray.chem.agilent.com/suredesign/index.htm). The percentages of bases aligning to target regions was calculated by dividing the number of aligned bases (with a mapping quality > 0) in bait regions, by the total number of aligned bases (also with a mapping quality > 0). "Bait", not "target", values from HsMetrics were used for on/off target calculations as these do not exclude low quality reads. "Target" values were used for coverage metrics as "bait" values were not available.

4 Demonstration of HeteroGenesis with w-Wessim

Heterogenesis was used to simulate a tumour, which was then sequenced by w-Wessim to create datasets of 100,000,000 read pairs per clone. These were aligned to the hg38 reference genome with BWA-MEM. Supplementary Fig. S7-S8 shows the germline and clone1 datasets viewed in IGV.

CNV events in region chr3:183260000-184110000	
Germline	copy=B, position= 183297265-183320357, number=6, order=20256
Clone1<br>(In addition to germline variants)	copy=A, position= 184039447-184084390, number=3, order=509940<br>whole-chromosome aneuploid event, copy=B, number=2, order=563034

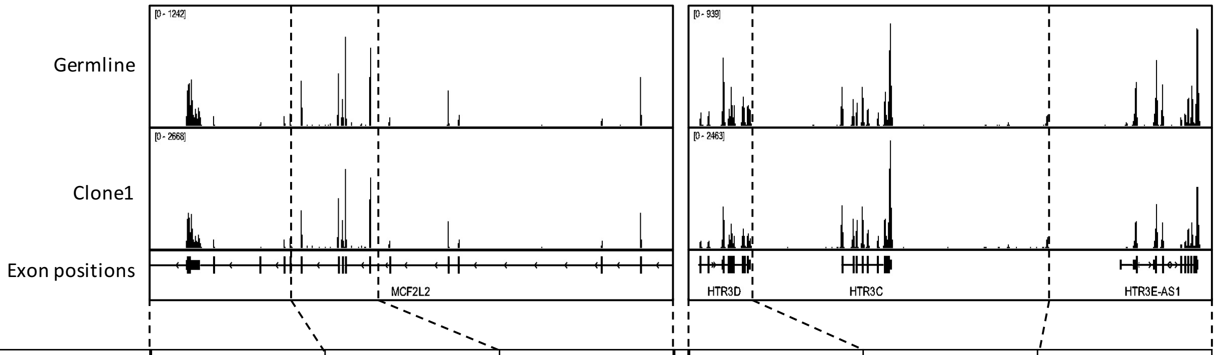

Position on chr3		183260000-183297264	183297265-183320356	183320357-183340000	184030000-184039445	184039446-184084390	184084391-184110000
Expected copy number	Germline	2	7	2	2	2	2
	Clone1	3	13	3	3	5	3
Read count	Germline	11064	12315	5433	5776	9105	9892
	Clone1	18391	24772	8782	9664	25664	16481
Read count/copy number	Germline	5532	1759	2717	2888	4552	4946
	Clone1	6130	1906	2927	3221	5133	5494
Ratio across regions	Germline	1	0.318	0.491	1	1.57	1.71
	Clone1	1	0.311	0.477	1	1.59	1.71

Supplementary Figure S7. Sequencing datasets for the germline and clone1 of a tumour simulated by HeteroGenesis and *in silico* sequenced by w-Wessim, viewed on IGV. Read counts for each region are divided by the expected copy number and then divided by the number of reads in the first region to get the ratio of reads between regions. Equal ratios across regions, between the germline and clone1 samples indicate appropriate modelling of copy numbers.

Examples of variants in region chr3:183260000-184110000	
Germline	copy=B, position=183310515, variant=C>T, order=19218<br>copy=B, position=183297265-183320357, number=6, order=20256<br>copy=B, position=183762321, variant=TGTTG>T, order=108654
Clone1 (In addition to germline variants)	copy=B, position=183311543, variant=C>T, order=440382<br>copy=A, position=184039447-184084390, number=3, order=509940<br>whole-chromosome aneuploid event, copy=B, number=2, order=563034<br>copy=B-2, position=183311639, variant=C>A, order=565763

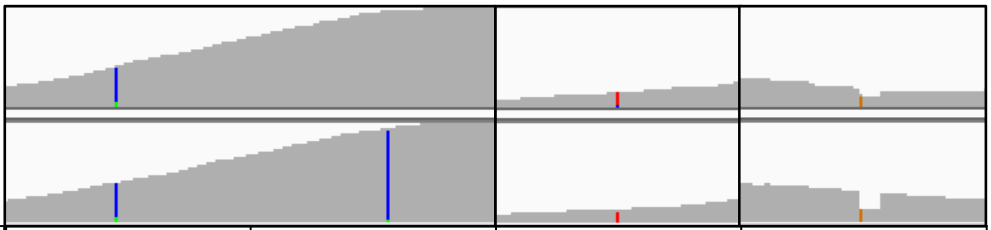

	Position on chr3	183311543	183311639	183310515	183762321
	Variant	C>A	C>A	C>T	TGTTG>T
Germline	Expected frequency	0.14286	0	0.85714	0.5
	Allele counts	C=347, A=54	C=924, T=1	C=9, T=42	T=7, TT=3(mis-mapped deletions), TGTTG=15-16
	Observed frequency	0.135	0	0.824	0.4
Clone 1	Expected frequency	0.15385	0.07692	0.92308	0.66667
	Allele counts	C=664, A=102	C=1673, A=96, G=1	C=11, T=82	T=25, TT=2(mis-mapped deletions), TGTTG=22-23
	Observed frequency	0.134	0.0542	0.882	0.54

Supplementary Figure S8. Examples of variants in sequencing datasets for the germline and clone1 of a tumour simulated by HeteroGenesis and *in silico* sequenced by w-Wessim, viewed on IGV.

5. References

1000 Genomes Project Consortium,T. 1000 G.P. *et al.* (2015) A global reference for human genetic variation. *Nature*, **526**, 68–74.

Baysan,M. *et al.* (2017) Detailed longitudinal sampling of glioma stem cells *in situ* reveals Chr7 gain and Chr10 loss as repeated events in primary tumor formation and recurrence. *Int. J. Cancer*, **141**, 2002–2013.

Broad Institute Picard Tools - By Broad Institute.

Cibulskis,K. *et al.* (2013) Sensitive detection of somatic point mutations in impure and heterogeneous cancer samples. *Nat. Biotechnol.*, **31**, 213–219.

Droop,A. *et al.* (2018) How to analyse the spatiotemporal tumour samples needed to investigate cancer evolution: A case study using paired primary and recurrent glioblastoma. *Int. J. Cancer*, **142**, 1620–1626.

Durbin,R.M. *et al.* (2010) A map of human genome variation from population-scale sequencing. *Nature*, **467**, 1061–1073.

Ewing,A.D. *et al.* (2015) Combining tumor genome simulation with crowdsourcing to benchmark somatic single-nucleotide-variant detection. *Nat. Methods*, **12**, 623–630.

Fan,Y. *et al.* (2016) MuSE: accounting for tumor heterogeneity using a sample-specific error model improves sensitivity and specificity in mutation calling from sequencing data. *Genome Biol.*, **17**, 178.

Gerlinger,M. *et al.* (2014) Genomic architecture and evolution of clear cell renal cell carcinomas defined by multiregion sequencing. *Nat. Genet.*, **46**, 225–233.

Hosny,A. (2017) NabaviLab/VarSimLab.

Hu,Y. *et al.* (2013) Tumor-Specific Chromosome Mis-Segregation Controls Cancer Plasticity by Maintaining Tumor Heterogeneity. *PLoS One*, **8**, e80898.

Ivakhno,S. *et al.* (2017) tHapMix: simulating tumour samples through haplotype mixtures. *Bioinformatics*, **33**, 280–282.

Kandoth,C. *et al.* (2013) Mutational landscape and significance across 12 major cancer types. *Nature*, **502**, 333.

Kent,W.J. (2002) BLAT--the BLAST-like alignment tool. *Genome Res.*, **12**, 656–64.

Kim,S. *et al.* (2013) Wessim: a whole-exome sequencing simulator based on in silico exome capture. *Bioinformatics*, **29**, 1076–1077.

Krijgsman,O. *et al.* (2014) Focal chromosomal copy number aberrations in cancer—Needles in a genome haystack. *Biochim. Biophys. Acta - Mol. Cell Res.*, **1843**, 2698–2704.

Li,H. (2013) Aligning sequence reads, clone sequences and assembly contigs with BWA-MEM. *arXiv*.

McElroy,K.E. *et al.* (2012) GemSIM: general, error-model based simulator of next-generation sequencing data. *BMC Genomics*, **13**, 74.

McPherson,A. *et al.* (2016) Divergent modes of clonal spread and intraperitoneal mixing in high-grade serous ovarian cancer. *Nat. Genet.*, **48**, 758–767.

Mills,R.E. *et al.* (2006) An initial map of insertion and deletion (INDEL) variation in the human genome. *Genome Res.*, **16**, 1182–90.

Mu,J.C. *et al.* (2015a) VarSim: a high-fidelity simulation and validation framework for high-throughput genome sequencing with cancer applications. *Bioinformatics*, **31**, 1469–1471.

Mu,J.C. *et al.* (2015b) VarSim: a high-fidelity simulation and validation framework for high-throughput genome sequencing with cancer applications. *Bioinformatics*, **31**, 1469–1471.

Qin,M., Liu,B., Conroy,J.M., Morrison,C.D., Hu,Q., *et al.* (2015) SCNVSim: somatic copy number variation and structure variation simulator. *BMC Bioinformatics*, **16**, 66.

Qin,M., Liu,B., Conroy,J.M., Morrison,C.D., Marth,G., *et al.* (2015) SCNVSim: somatic copy number variation and structure variation simulator. *BMC Bioinformatics*, **16**, 66.

Salcedo,A. *et al.* (2018) Creating Standards for Evaluating Tumour Subclonal Reconstruction. *bioRxiv*, 310425.

Semeraro,R. *et al.* (2018) Xome-Blender: A novel cancer genome simulator. *PLoS One*, **13**, e0194472.

Sherry,S.T. *et al.* (2001) dbSNP: the NCBI database of genetic variation. *Nucleic Acids Res.*, **29**, 308–11.

Sottoriva,A. *et al.* (2013) Intratumor heterogeneity in human glioblastoma reflects cancer evolutionary dynamics. *Proc. Natl. Acad. Sci. U. S. A.*, **110**, 4009–14.

Tan,R. *et al.* (2014) An Evaluation of Copy Number Variation Detection Tools from Whole-Exome Sequencing Data. *Hum. Mutat.*, **35**, 899–907.

Watkins,T.B.K. and Schwarz,R.F. (2018) Phylogenetic Quantification of Intratumor Heterogeneity. *Cold Spring Harb. Perspect. Med.*, **8**, a028316.

Xi,R. *et al.* (2011) Copy number variation detection in whole-genome sequencing data using the Bayesian information criterion. *Proc. Natl. Acad. Sci. U. S. A.*, **108**, E1128-36.

Xia,L.C. *et al.* (2018) SVEngine: an efficient and versatile simulator of genome structural variations with features of cancer clonal evolution. *Gigascience*, **7**.

Xia,Y. *et al.* (2017) Pysim-sv: a package for simulating structural variation data with GC-biases. *BMC Bioinformatics*, **18**, 53.
